# ARN: analysis and prediction by adipogenic professional database

**DOI:** 10.1186/s12918-016-0321-0

**Published:** 2016-08-08

**Authors:** Yan Huang, Li Wang, and Lin-sen Zan

**Affiliations:** College of Animal Science and Technology, Northwest A&F University, Yangling, Shaanxi 712100 China

**Keywords:** Adipogenesis, Regulation, Database, Analysis

## Abstract

Adipogenesis is the process of cell differentiation by which mesenchymal stem cells become adipocytes. Extensive research is ongoing to identify genes, their protein products, and microRNAs that correlate with fat cell development. The existing databases have focused on certain types of regulatory factors and interactions. However, there is no relationship between the results of the experimental studies on adipogenesis and these databases because of the lack of an information center. This information fragmentation hampers the identification of key regulatory genes and pathways. Thus, it is necessary to provide an information center that is quickly and easily accessible to researchers in this field. We selected and integrated data from eight external databases based on the results of text-mining, and constructed a publicly available database and web interface (URL: http://210.27.80.93/arn/), which contained 30873 records related to adipogenic differentiation. Then, we designed an online analysis tool to analyze the experimental data or form a scientific hypothesis about adipogenesis through Swanson’s literature-based discovery process. Furthermore, we calculated the “Impact Factor” (“IF”) value that reflects the importance of each node by counting the numbers of relation records, expression records, and prediction records for each node. This platform can support ongoing adipogenesis research and contribute to the discovery of key regulatory genes and pathways.

**Electronic supplementary material:**

The online version of this article (doi:10.1186/s12918-016-0321-0) contains supplementary material, which is available to authorized users.

## Background

Adipose tissue is an important site for lipid storage, energy homeostasis, and whole-body insulin sensitivity. It is important to understand the mechanisms involved in adipose tissue development. Growth of adipose tissue is the result of differentiation of new fat cells from precursor cells [1]. It is obvious that adipogenesis is not a single gene trait, but is determined by a number of genes and their encoded proteins [2]. Therefore, researchers need a professional comprehensive knowledge database including related genes, proteins, properties, biological processes, and environmental factors in accordance with their determined or predicted relations in the literature to assist researchers in understanding adipogenic differentiation from the perspective of systems biology.

After obtaining a large amount of data and information related to fat, a key element is linking the extracted information together to form new facts or hypotheses to be explored further by more conventional means of experimentation [3]. Swanson developed and implemented a novel tool to mine the existing knowledge base for unreported or underreported relationships, and highlighted previously published but neglected hypotheses, a process known as literature-based discovery [4]. This process functions by connecting two seemingly unrelated findings [5]. This and implemented a novel tool to mine the existing knowledge and easily accessible to researchers in this field. Conclusive proof, the discovery is, in itself, very helpful to uncover previously unknown relationships [6]. Furthermore, it can help investigators access context and mine knowledge that might not be revealed using a traditional search.

In the present study, we constructed a molecular regulatory network of adipogenesis based on text-mining and manual examination, and then screened the data of four external databases according to the network, which produced more than 10 000 prediction results out of >1 × 10^6^ interaction records (Table 1). Moreover, we designed an online analysis tool according to the theory of “literature-based discovery”, which provides a new approach for researchers to analyze data and form hypotheses. Ultimately, we explored the possibility of using a large amount of accumulated data to promote future research and practices.

**Table 1:** External databases

No.	URL	PMID	Relation type	Total records	Records in ARN
1	http://www.grnpedia.org/trrust/ [45]	26066708 [46]	TFs-Targets	8215	3538
2	http://www.pazar.info/ [47]	18971253 [48]	TFs-Targets	6869	1080
3	http://mirgate.bioinfo.cnio.es [49]	25858286 [50]	miRs-Targets	38810	8069
4	http://thebiogrid.org/ [51]	16381927 [52]	Protein-Protein	1066335	182

## Construction and content

**Fig. 1:**
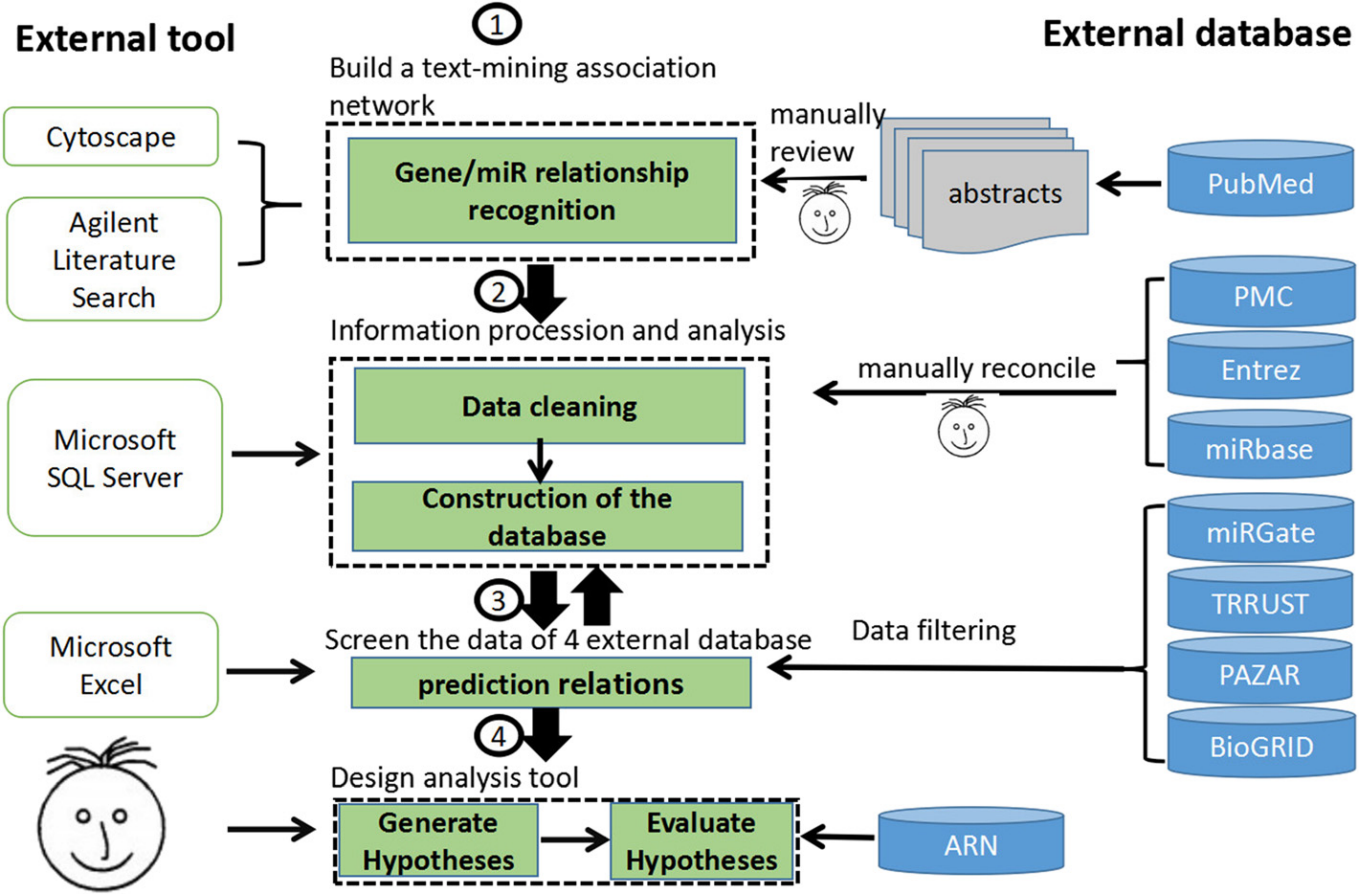
Database construction pipeline. Database construction was performed as four major steps. The whole pipeline is based on PubMed-derived abstracts related to adipogenesis research

The Adipogenic Regulation Network (ARN) Database aims to provide a high-quality collection of genes, microRNAs, and their relations implicated in the regulation of adipogenesis, which has been reviewed by experts in the field. The data collection and processing steps are illustrated in Fig. 1. The workflow included four major steps. Step one: build a text-mining association network using the Agilent Literature Search plugin [7]. Step two: information processing and analysis. Step three: screen the data of four external databases (Table 1) according to the network. Step four: design analysis tool.

### Information mining and manual review

For the literature search, we established a set of queries by entering 47 key genes in adipogenesis [7] with simultaneous input contexts ‘adipo* differen*’, which is short for “adipocyte differentiation”. The query set was submitted one at a time to PubMed by Agilent Literature Search. The resulting documents were retrieved, parsed into sentences, and analyzed for known interaction terms such as ‘binding’ or ‘activate’. Agilent Literature Search uses a lexicon set to define gene names (concepts) and aliases, drawn from Entrez Gene, and interaction terms (verbs) of interest. An association was extracted from every sentence containing at least two concepts and one verb. Associations were then converted into interactions with corresponding sentences and source hyperlinks, and added to a Cytoscape network [8]. The last download of abstracts was executed on 29 October 2015. In total, 9908 PubMed abstracts were obtained and served as the initial corpus for further processing.

The literature mining method has problems including ‘term variation’ and ‘term ambiguity’ [9]. Term variation originates from the ability of a natural language to express a single concept in a number of ways. For example, in biomedicine, there are many synonyms for proteins, enzymes, and genes. Having six or seven synonyms for a single concept is not unusual in this domain [10]. In the ARN database, we unified a gene as the official gene symbol. Term ambiguity occurs when the same term is used to refer to multiple concepts. For example, the term “fat” can be a noun or an adjective for “obese”. The two terms are often used in biomedical literature. Searching for “fat” in PubMed returned 187888 results. We found that fat was also used to name a gene or as a universal symbol. Therefore, it was necessary to carry out a manual examination of the results of literature mining to delete the wrong results. During this process, we removed most of the 9908 PubMed abstracts, and only 1449 remained.

### Information processing and analysis

**Fig. 2:**
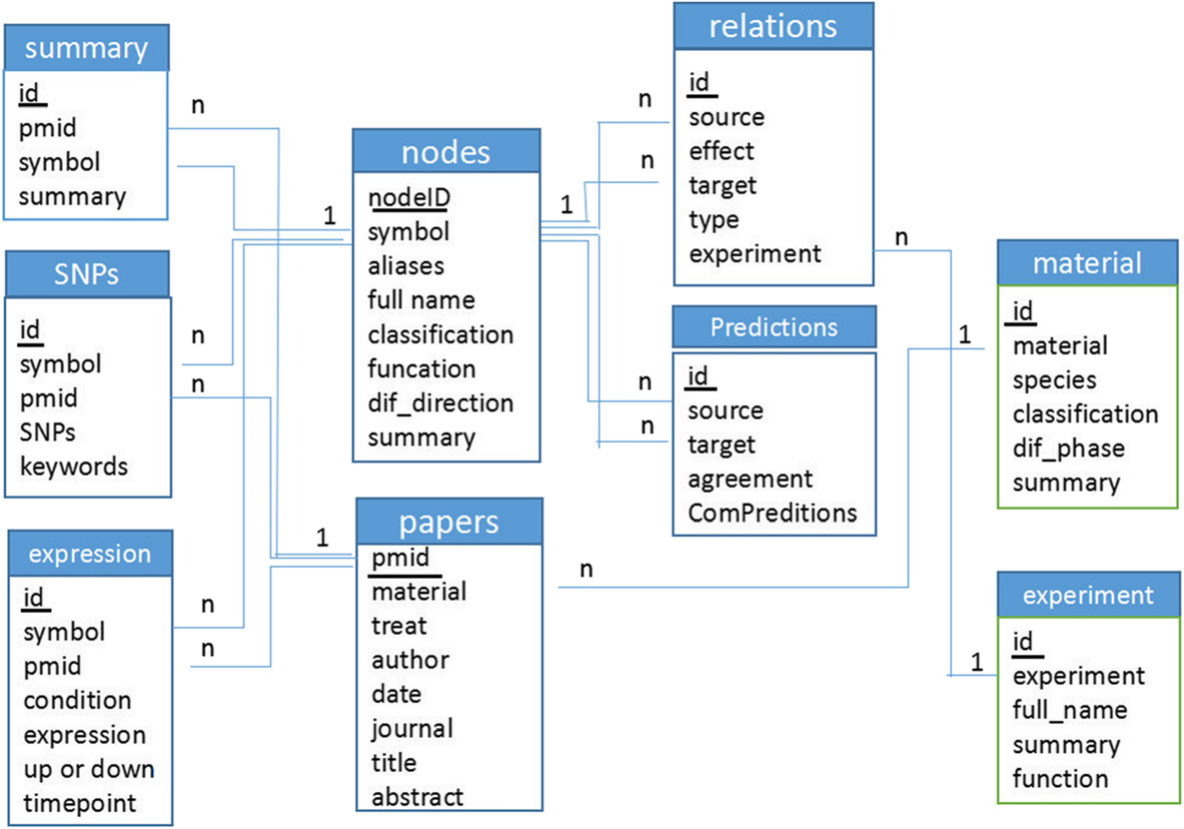
Table structure of the database. Table “Node” includes the information of the nodes (genes and microRNAs) that regulate adipogenesis. Table “Papers” includes the information of papers about adipogenesis. Table “Summary” includes the summary of the nodes. Table “SNPs” includes information about the SNPs of nodes. Table “Expression” includes the information about expression of nodes in different conditions. Table “Relations” includes the information about the relations of nodes. Table “Material” includes information about the experimental materials in papers. Table “Experiment” includes information about the experiments that were used to verify the relations of nodes

During the manual annotation and analysis step, information about experimental settings, node classification, function, and adipogenic impact was marked. For each paper in the ARN database, the experimental setting included the experimental procedure and names of cell lines, and the kind of samples was also classified. To store and access the collected information of adipogenesis regulatory networks, we implemented a database and a user-friendly web interface. The ARN database is a Microsoft SQL Server relational database. The table structure of the database is illustrated in Fig. 2, and its complete content is shown in Additional file 1: Table S1.

### Screening the data of four external databases

**Fig. 3:**
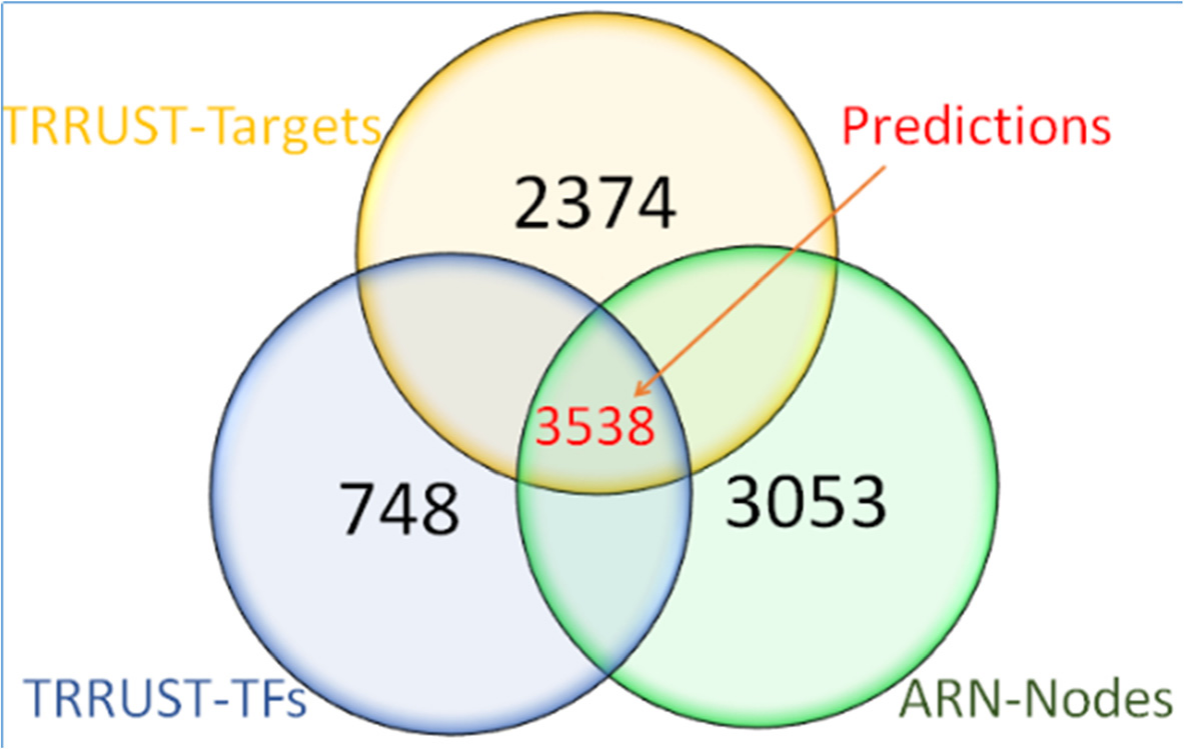
Venn diagram to represent the overlap between TRRUST and ARN databases

Sometimes, the dormant value can only be revealed by combining one dataset with another, perhaps a very different dataset. For example, we obtained 748 transcription factors and their 2347 targets according to 8215 records in transcriptional regulatory relationships unravelled by sentence-based text-mining (TRRUST) database. Next, we concluded that 3538 TF-Target records in TRRUST may be associated with adipogenesis according to the 3053 nodes in the ARN database (Fig. 3). Using the same method, we screened the other three databases. Their basic information is shown in Table 1. In the future, when a new professional database appears, we could rapidly add data associated with adipogenic differentiation to the ARN database according to this method.

### Design of the analysis tool

Our interest in text-based scientific discovery led us to the development of the ARN-Analysis tool. Because we envision text-based discovery as a human-centered activity, our goal has been to codify a practical tool that assists a biomedical researcher in formulating and initially testing hypotheses [11].

#### Generating hypotheses

As shown in Fig. 2 and Additional file 1: Table S1, the information is structured in the ARN database. Therefore, the discovery question is user generated on which subject the user wants to obtain new knowledge. Additionally, the filtering and selection of interesting B- or C-concepts is user dependent. Interesting in this case means interesting according to the current knowledge and goals of the user. It is the user who will have to make an interpretation of the computer-suggested list of possible results. Finally, the intersection of two or more result sets can be obtained by the user, which is likely to be hypotheses.

#### Testing hypotheses

Once we have many hypotheses, which may be obtained by the ARN-Analysis tool, we may want to determine which is the most important by initially screening out nodes that are highly correlated with adipogenesis. For this purpose, we calculated the “IF” value for each node with the following formula:

$$ \mathrm{IF}\left(\mathrm{i}\right)=\left[\mathrm{R}\mathrm{i}/\mathrm{Rmax}+\mathrm{E}\mathrm{i}/\mathrm{Emax}+\mathrm{Pi}/\mathrm{Pmax}\right]/3\times 100\% $$

In this formula, IF (i) is the effect of node i on the differentiation of fat. Ri is the number of relationships of node i, Rmax is the number of relationships of node r-max that has the most relations; Ei is the number of expression records of node i. Emax is the number of expression records of node e-max that has the most expression records; Pi is the number of prediction records of node i. Pmax is the number of prediction records of node p-max that has the most prediction records. All values are updated with the database, so the information they contain is comprehensive and timely.

## Utility

### Basic information of the ARN database

Currently, the database contains 3054 nodes (genes and microRNAs), 1807 relation records, 1141 summary records, 10675 expression records, and 43 review images associated with adipogenesis according to 1457 papers. Among the 3054 nodes in the ARN database, we determined 12869 possible relationships sourced from miRGate, TRRUST, BioGRID and PAZAR.

The database can be searched using a web interface (http://210.27.80.93/arn/) [12] with three possible input forms depending on the user’s research focus. For gene searches, Entrez GeneID and official gene symbols are accepted. MicroRNAs require the names of mature microRNA sequences (e.g., mirn143). The literature requires the PubMed PMID (see Additional file 2: Handbook of ARN, Example 1). We provide the node, maps, literature, and expression pages for different kinds of information. Users can select their requested entry and the results page is displayed.

### Correlations between databases

**Fig. 4:**
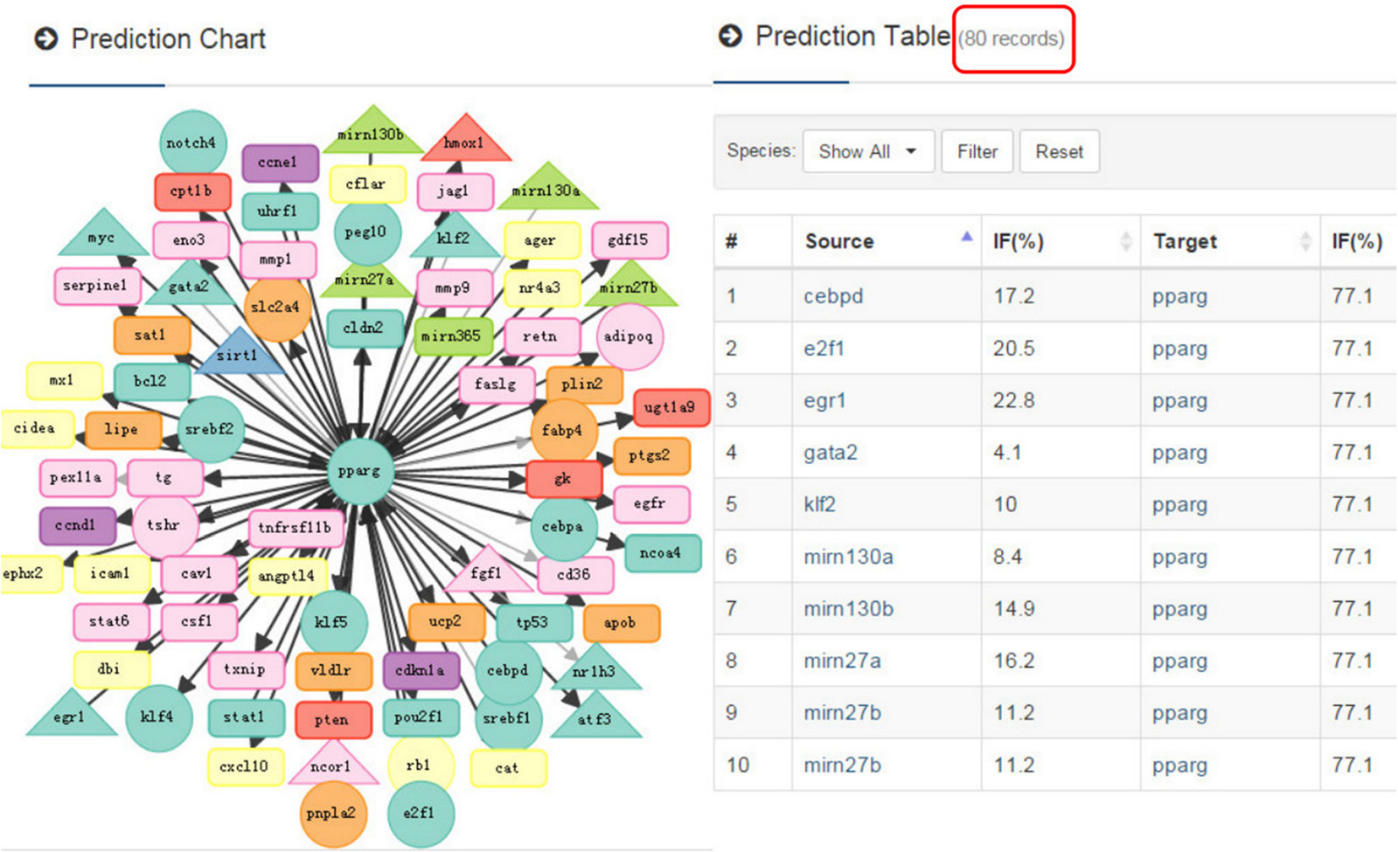
Part of the prediction relations of PPAR-gamma. Nodes of interest can be chosen by clicking on their node page. Data in this diagram are only part of the prediction results. For the whole data set, see the ARN database

A total of 12869 predicted results were obtained by screening the data of four external databases. They are the product of correlations between the ARN database and external databases. As shown in Fig. 4, in the node page, the prediction relations of each node are plotted as a graph, and the relevant information is provided in the form at the same time.

### ARN-Analysis is a professional analysis tool for the study of adipogenesis

**Fig. 5:**
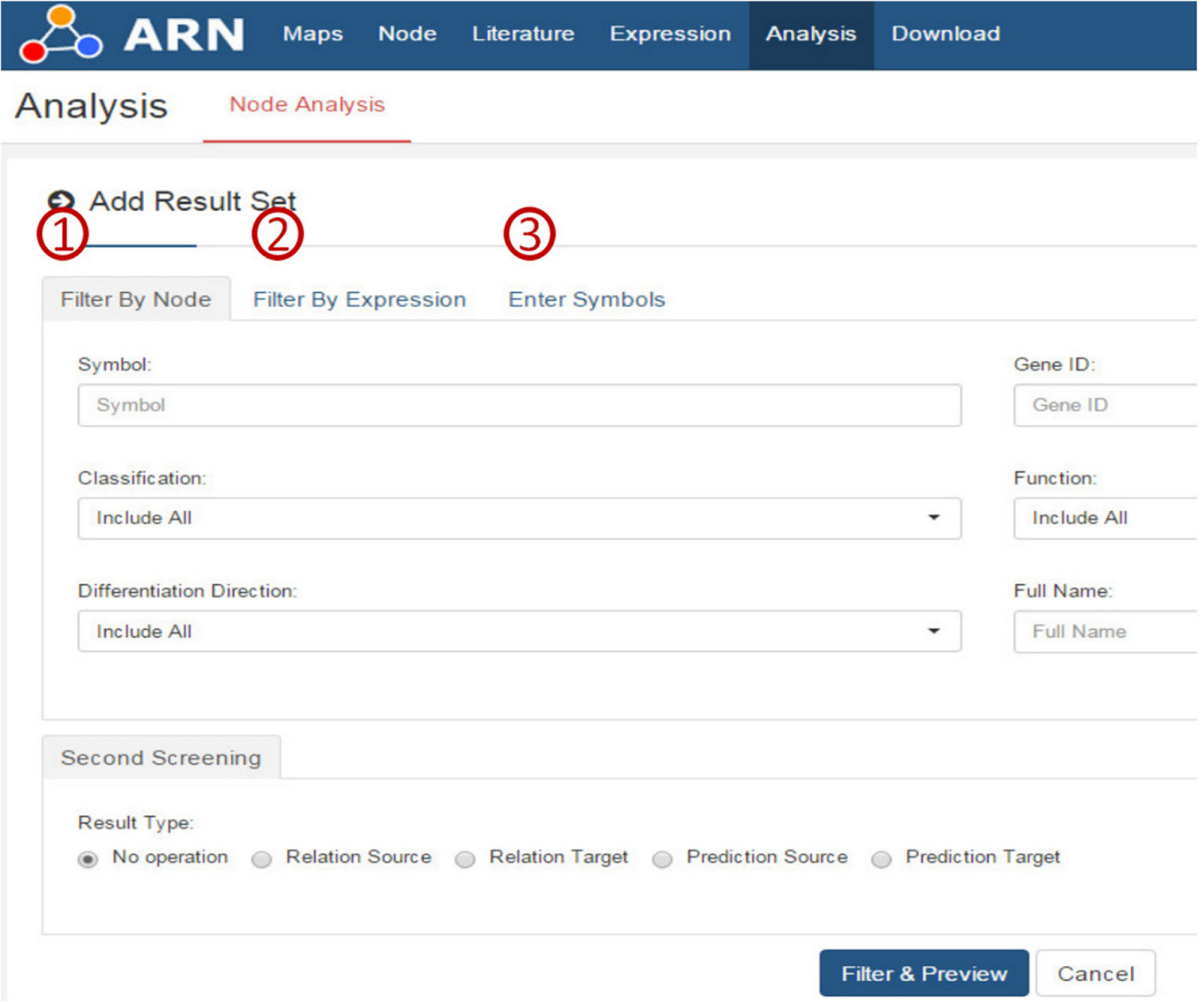
Three analytical models of the ARN-Analysis tool

The analysis page was constructed to screen functional genes and microRNAs based on all the information in the ARN database. Figure 5 shows the three analytical models that we designed for the needs of researchers.

**Fig. 6:**
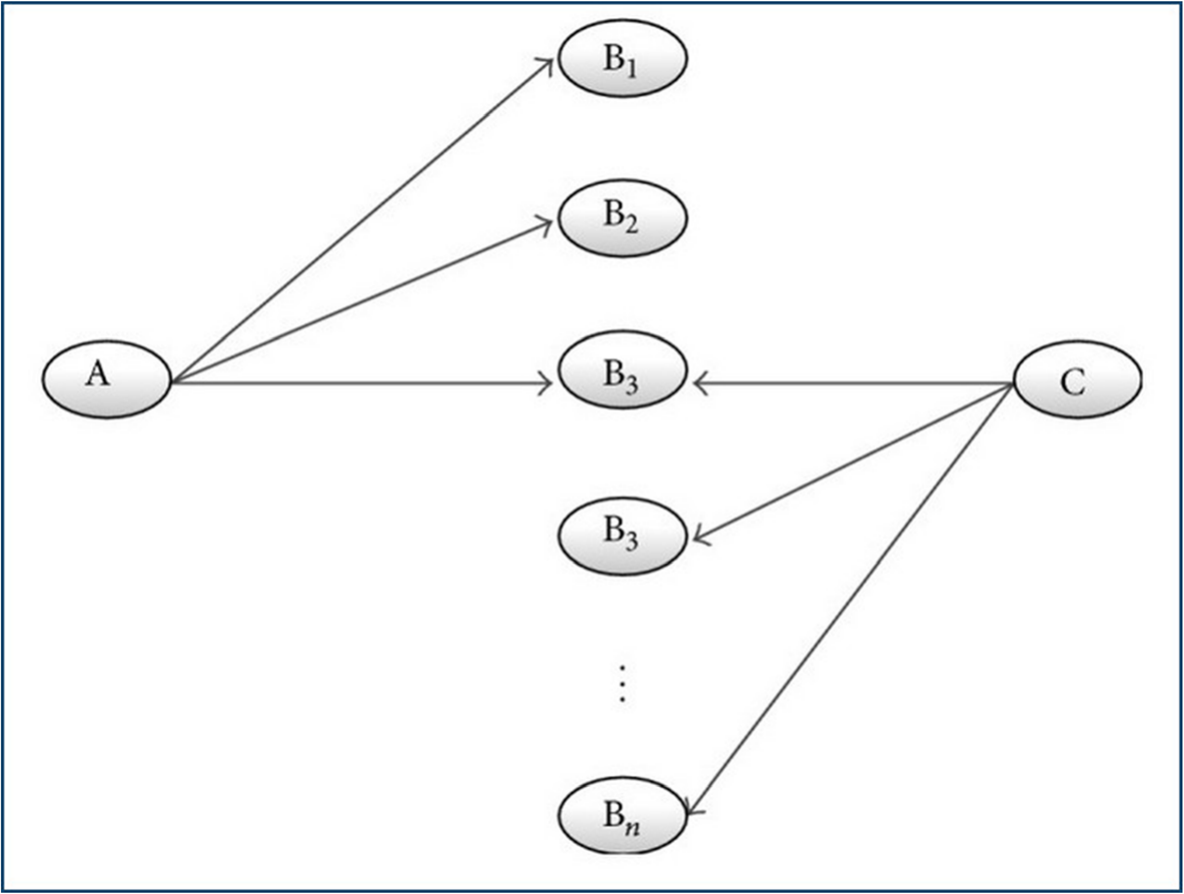
Discovery process as defined by Weeber et al [53]. The process is a two-way discovery process that starts from A and C simultaneously, and then finds the intersection B

For nodes, researchers can use the “filter by nodes” page, according to the node type, its effects on adipogenesis, to screen node itself, its targets, its source nodes, its predicted source nodes, or its predicted targets. For example, Kim et al. reported that overexpression of mirn21 enhances the differentiation of adipocytes [13]. Therefore, we hypothesized that mirn21 promotes the formation of fat by reducing the expression of proteins that inhibit adipogenesis. Specifically, we needed to discover which mirn21 target genes may inhibit the formation of fat. As shown in Fig. 6, we defined “A” as “adipogenesis”, “B” as “gene”, “C” as “mirn21”, and started from A and C simultaneously, searched for the intersection of the two result sets, and found the answer. The operational procedure was as follows. In the “ARN-Analysis” page, we first entered “mirn21” in the “Symbol” text box. Second, we selected “Prediction Target” in the “Second Screening” options. Third, we clicked on “Filter & Preview”, and there were 60 genes in the result set. We then clicked “Save” to save the “C to B” result set. To obtain the “A to C” result set, we first clicked “Add Result Set” and then selected “Pro-adipogenesis” in the “Differentiation Direction” options. Here, we obtained 173 genes in the result set and then clicked "Save" to save the “A to B” result set. Finally, we clicked “Analysis” and obtained the intersection of the two result sets, which was the answer. It suggested that mirn21 may promote adipogenesis by inhibiting two pro-adipogenesis proteins, NFAT5 and Reck.

**Fig. 7:**
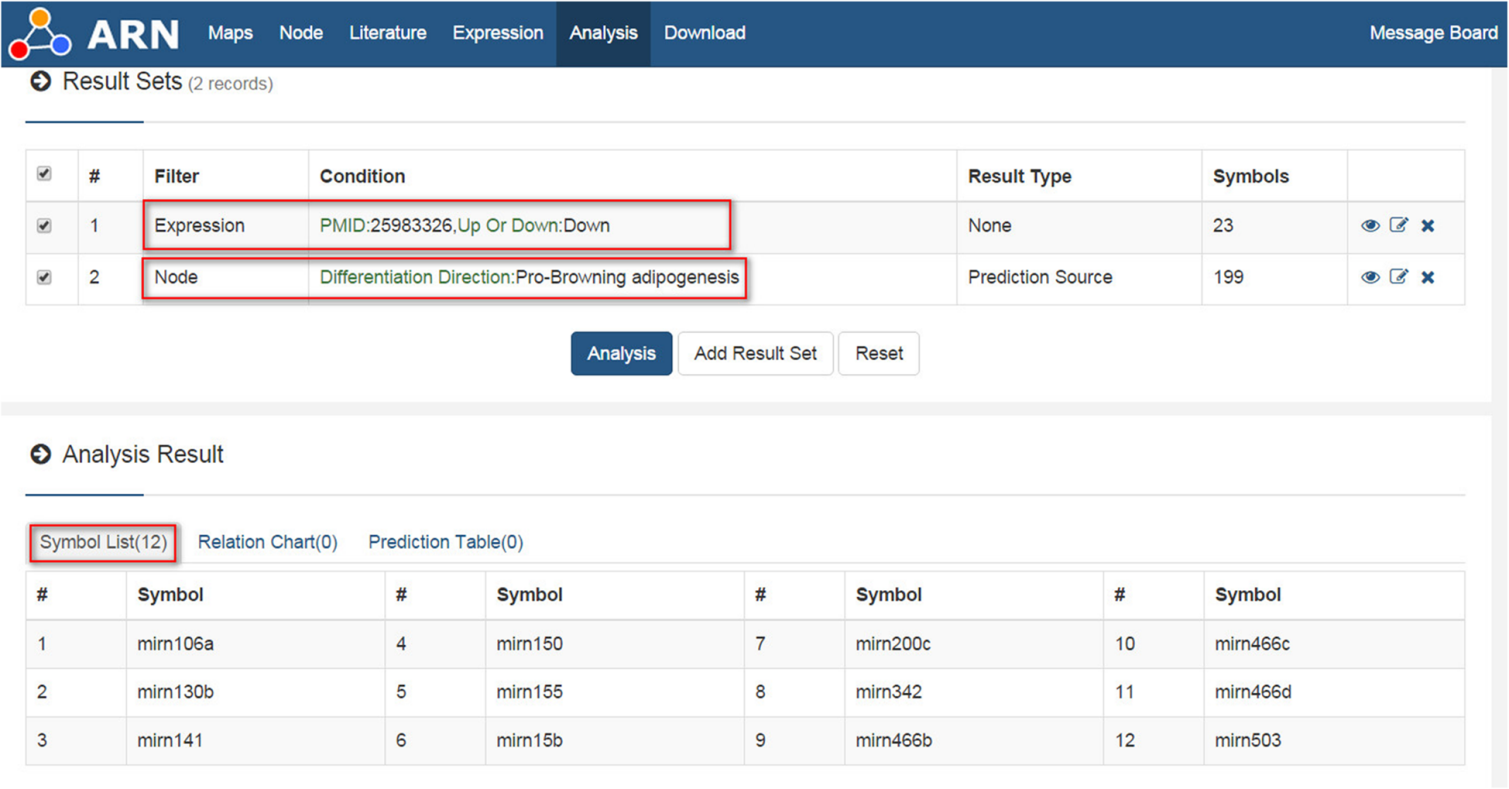
Example of “Filter By Expression”

**Fig. 8:**
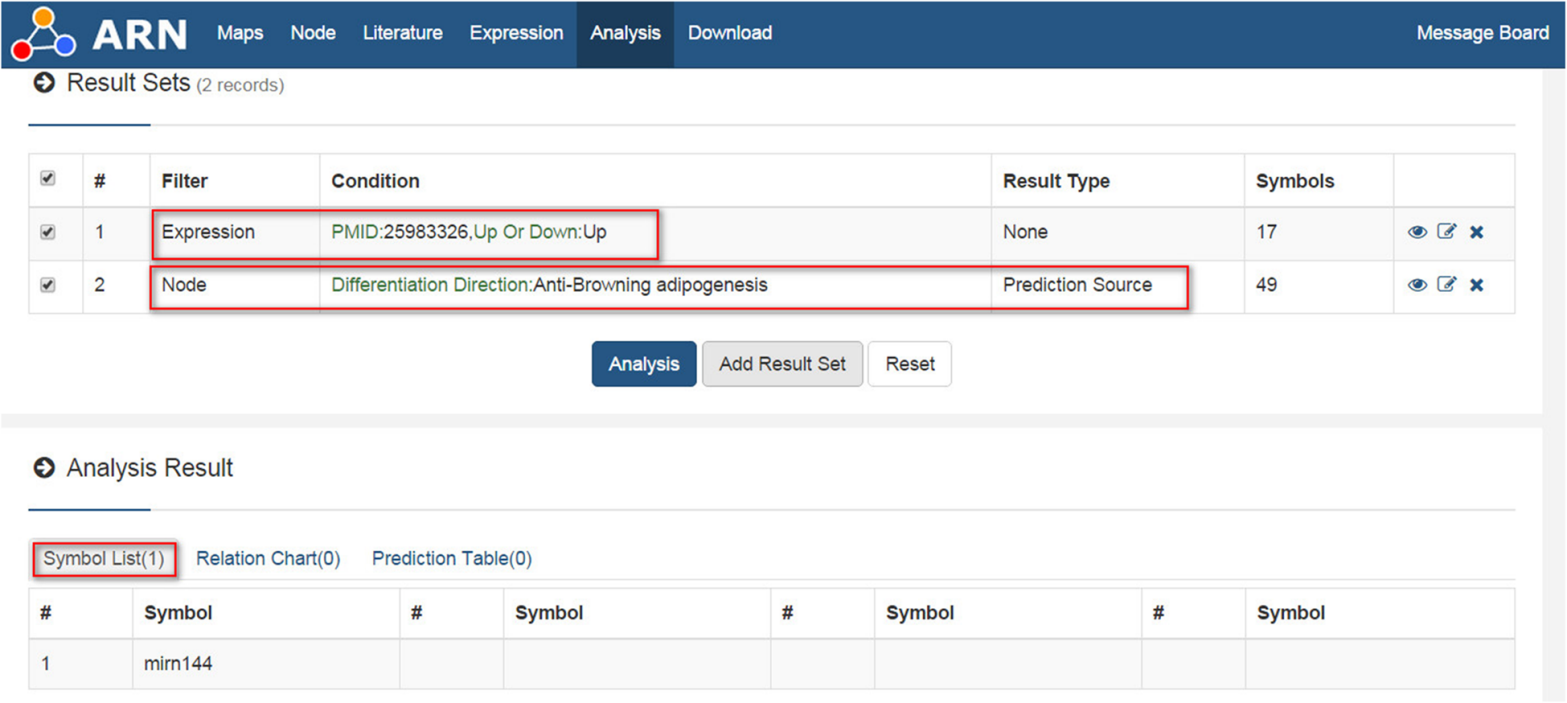
Example 2 of “Filter By Expression”

In addition, because there are thousands of differentially expressed nodes in the result sets of many high-throughput articles, usually the author will focus on analysis of a few of them, but most of the remaining nodes may contain important information. Therefore, we designed “Filter By Expression”. Additional file 3: Table S2 contains all of the throughput article PMIDs in the ARN database. Researchers can analyze each of the result sets according to Additional file 3: Table S2. Researchers can also first obtain the throughput article’s PMID in the “ARN-literature page” (see Additional file 2: Handbook of ARN, Example 2), and then use "Filter By Expression" to analyze it. For example, we chose one paper (PMID 25983326) in the “ARN-literature page” found by “advanced search”. This paper describes changes in microRNA expression of white and brown adipose tissues in cold-induced mice [14]. Cold stimulation contributes to the formation of brown fat [15]. Therefore, we hypothesized that some up-regulated microRNAs may inhibit “Anti-browning adipogenesis” proteins, while some down-regulated miRNAs may inhibit “Pro-browning adipogenesis” proteins. Next, we performed the analysis in ARN as shown in Figs. 7 and 8. The results showed that one up-regulated microRNA and 12 down-regulated microRNAs were consistent with our hypothesis. By further analysis in ARN, we obtained the results shown in Table 2.

**Table 2:** Changes in microRNA expression of white and brown adipose tissues in cold-induced mice

Change	miRNA	Target
Up-regulated	mirn144	notch1
Down-regulated	mirn106a	Rb1
	mirn130b	Klf1
		pparg
		Prkaa1
	mirn141	esrrg
		Klf11
	mirn140	Tp53
	mirn155	cebpb
		Socs1
	mirn15b	apln
		Med1
	mirn200c	esrrg
		Klf11
	mirn342	Bmp7
	mirn466b	Ppargc1a

**Fig. 9:**
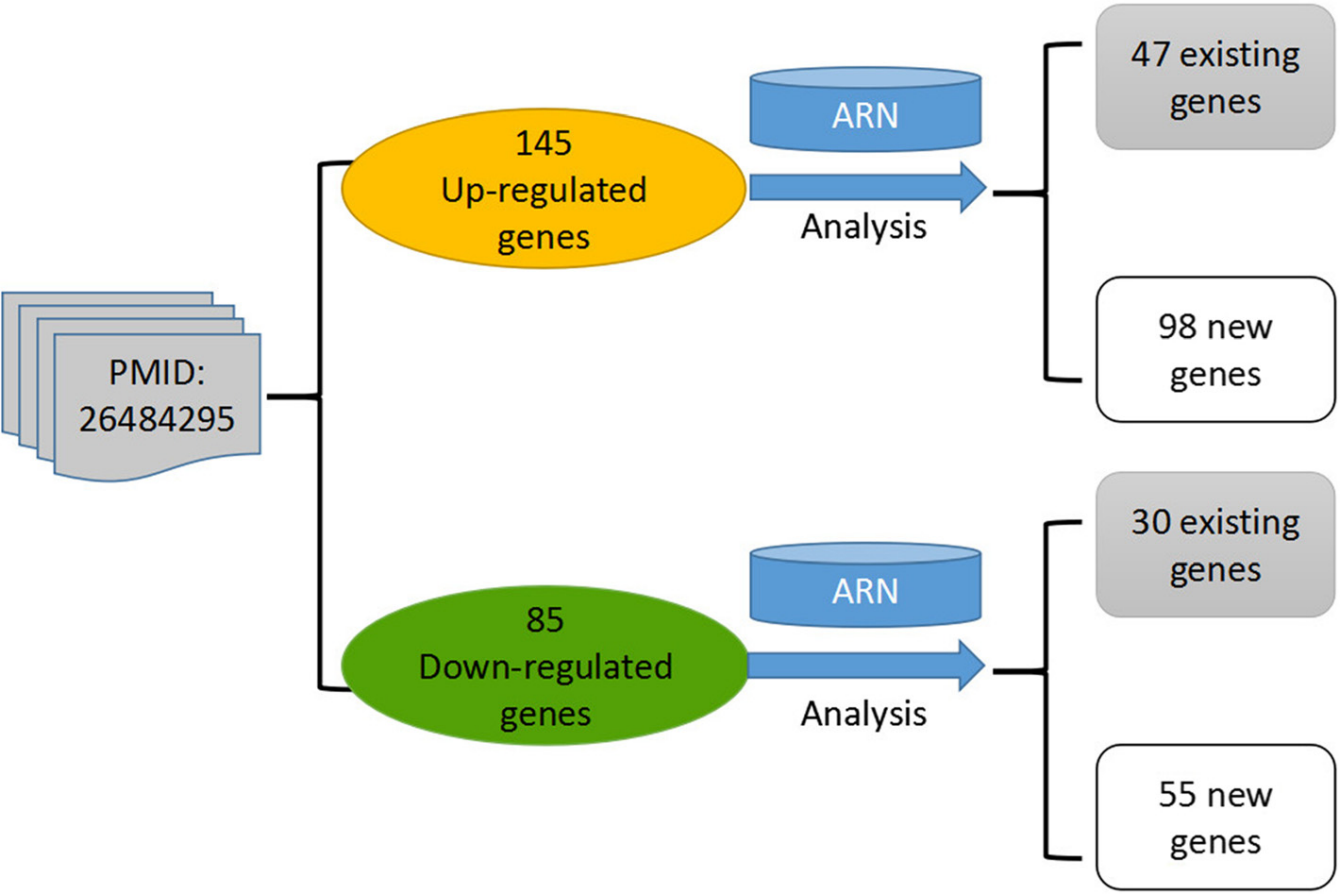
Analysis of a high-throughput data set of gene expression

Furthermore, some researchers need to analyze the result set of their experiment. Therefore, we designed “Enter Symbols”, which allows users to enter a series of genes or microRNAs for analysis. When Xiaoxi et al. performed transcriptome profiling of white adipose tissue in a mouse model for 15q duplication syndrome, they found 145 significantly up-regulated and 85 significantly down-regulated genes [16]. We entered them separately into the ARN-Analysis user-defined input box (see Additional file 2: Handbook of ARN, Example 5). The results of the analysis are shown in Fig. 9: Among the 145 up-regulated genes, 47 nodes were recorded in the ARN database and 98 nodes were newly discovered. Among the 85 down-regulated genes, 30 nodes were recorded in the ARN database and 55 nodes were newly discovered. Combined with other information about the known nodes in the database, we can construct hypotheses, design experiments, and perform further research.

### Scoring function of the ARN database

**Fig. 10:**
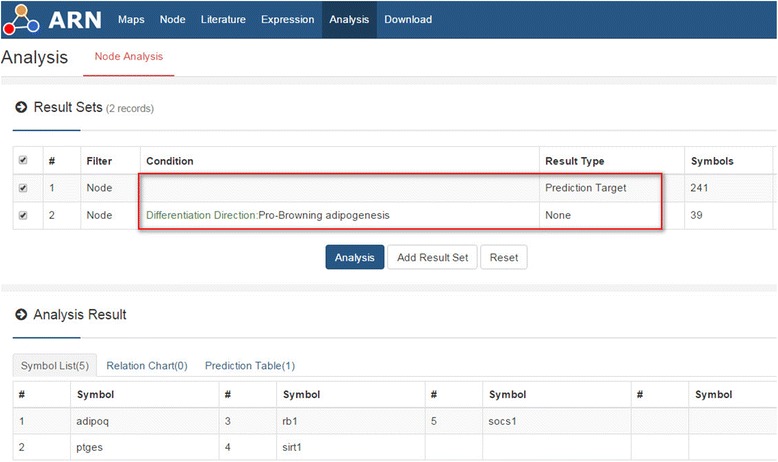
Screening the intersection between targets of Sp1 and pro-browning adipogenesis genes. The red box indicates the screening condition. See Additional file 2: Handbook of ARN, Example 2 for detailed information

“IF” of the node is obtained from the latest data in the ARN database. When we add a new node or information to the database, the IF of all nodes associated with it will be changed accordingly. This process ensures the timeliness and completeness of the information obtained by researchers. On the home page of the database, we provided the "Link Number" top 50 "hub" nodes. In Table 3, we compared it with the 50 nodes with the maximum “IF” value. Because the IF value indicates the importance of the node to the formation of fat, we assumed that the difference between the two included some new “hub” nodes that may contain certain regulatory relationships that remain to be verified. Using “sp1” as an example, by searching the database node page, we found that expression of the sp1 gene was significantly higher in the liver of cold-induced mice [17]. Therefore, we hypothesized that it is related to brown adipogenesis. Next, we carried out the analysis as shown in Fig. 10. The results suggested that SP1 may regulate the formation of brown fat by regulating “adipoq” [18], “ptges” [19], “rb1”, “sirt1” [20], or “socs1” [21] (see Additional file 2: Handbook of ARN, Example 2). Researchers can design experiments to verify the results. The same method can also be used to analyze the other top 50 nodes.

**Table 3:** IF values of the top 50 nodes in the ARN database

IF no.	Symbol	Relation no.	IF no.	Symbol	Relation no.
1	pparg	1	26	egr1	
2	cebpa	4	27	ccnd1	
3	cebpb	2	28	fos	
4	nfkb1	9	29	mirn185	
5	sp1		30	mirn17	
6	fabp4		31	mirn15a	
7	mirn149		32	hif1a	
8	stat3	6	33	mirn155	
9	runx2	3	34	klf4	50
10	adipoq	17	35	mirn98	
11	rela		36	mirn335	
12	myc		37	mirn34a	
13	brca1		38	igf1	16
14	jun		39	ar	
15	srebf1	8	40	e2f1	
16	tp53		41	vdr	
17	mirn92a		42	cebpd	28
18	scd		43	stat1	
19	il6	46	44	ctnnb1	5
20	creb1	7	45	mirn29b	
21	mirnlet7b		46	mirn221	
22	tnf	11	47	mirn9	
23	mirn181a		48	mirn22	
24	mirn30a		49	mirn24	
25	lpl		50	mirn16	

IF No. indicates the ranking of the nodes’ IF in the database; Relation No. indicates the ranking of the counts of relations of nodes in the database

## Discussion

### Target control of adipogenesis genes

**Fig. 11:**
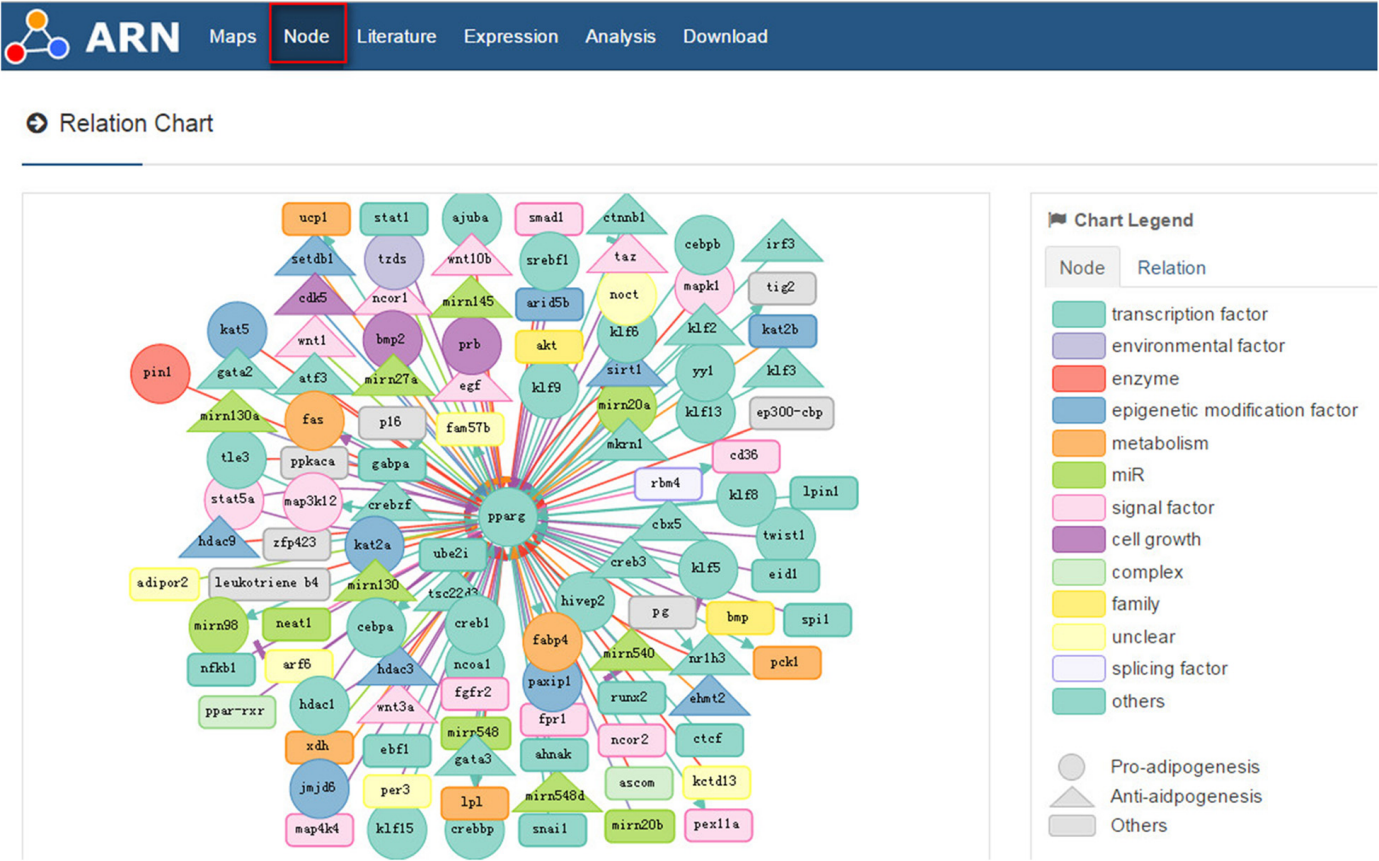
Relation chart of PPAR-gamma. Data in this image are only part of the relation information. For the whole data set, see the ARN database

Target control is controlling a subset of target nodes (or a subsystem), which is essential for the system’s mission pertaining to a selected task [22]. Only when we know all the relations of a node can we then know how to control it. The ARN database provides an overview of each node in the adipogenesis regulation network. As shown in Fig. 11 for the node PPAR-gamma, epigenetic modification of its chromatin [23–27], transcriptional regulation of its promoters [28–34], post-transcriptional regulation by microRNAs [35–38], and phosphorylation of its proteins by signaling factors [39, 40] from transcription initiation to the final degradation, this map describes its destiny. This information may help us to design an ideal strategy to control it. Using this information, we may be able to design target control approaches in the future.

### Deficiencies of the ARN database

In the process of adding the prediction relations to the ARN database through the external database, we found that the table structure of “Prediction” in Fig. 2 is inadequate. It lacks the relevant tags of the information source database, which obscures the specific sources of the prediction relations. In the future, we will correct this problem by upgrading the platform. In addition, for the IF calculation formula of each node at present, we determined the weights of Ri, Ei and Pi as 1/3. However, with continuous updating and improvement of the platform, the optimal weight of each influencing factor remains to be explored further.

### Future directions

The precursors of adipocytes, mesenchymal stem cells (MSCs), can also differentiate into osteoblasts, chondrocytes, and myoblasts. Understanding the factors that govern MSC differentiation has significant implications in diverse areas of human health from obesity to osteoporosis [41]. Therefore, we would like to add them to our network in the future. Moreover, recently, long-chain non-encoding RNA (lncRNA) was found to be involved in the regulation of adipogenic differentiation [42, 43]. These data must be added as soon as they are available. Furthermore, information on the institutions in the papers will soon be added. We are certain that this addition will promote the exchange of ideas, project cooperation, and resource sharing between institutions. We plan to update the database monthly to provide state-of-the-art knowledge and keep track of improvements in the field. All recently added data will be displayed separately on the corresponding page.

## Conclusions

The ARN database will serve as a platform for information and hypothesis generation for the research community, which will facilitate uncovering the complexity of adipogenesis-related mechanisms, pathways, and processes.

## Availability and requirements

Project name: ARNdbProject. Home page: http://210.27.80.93/arn/. Operating system(s): Platform independent. Other requirements: Microsoft SQL Server,. NET and HTML5 for the Web interface. For interactive data visualization, we applied D3.

## Abbreviations

IF, Impact Factor; ARN, Adipogenic Regulation Network; TRRUST, transcriptional regulatory relationships unravelled by sentence-based text-mining; MSCs, mesenchymal stem cells; lncRNA, long-chain non-encoding RNA

## Additional files

Additional file 1: Table S1. All tables of the ARN database. (XLSX 3574 kb) Additional file 2: Handbook of ARN. (PDF 15192 kb) Additional file 3: Table S2. All of the throughput articles’ PMID in ARN database. (XLSX 511 kb)
