# Supplementary material for: ARN: analysis and prediction by adipogenic professional database
Source: BMC Syst Biol. 2016 Aug 8;10:57. doi: 10.1186/s12918-016-0321-0 (PMC4977645; doi:10.1186/s12918-016-0321-0)
Supplement: Additional file 2: — Handbook of ARN. (PDF 15192 kb) [file 12918_2016_321_MOESM2_ESM.pdf]

Handbook of ARN database

http://210.27.80.93/arn/

ARN

Menu

Contents of ARN

Definitions

Examples

Contents of ARN Database

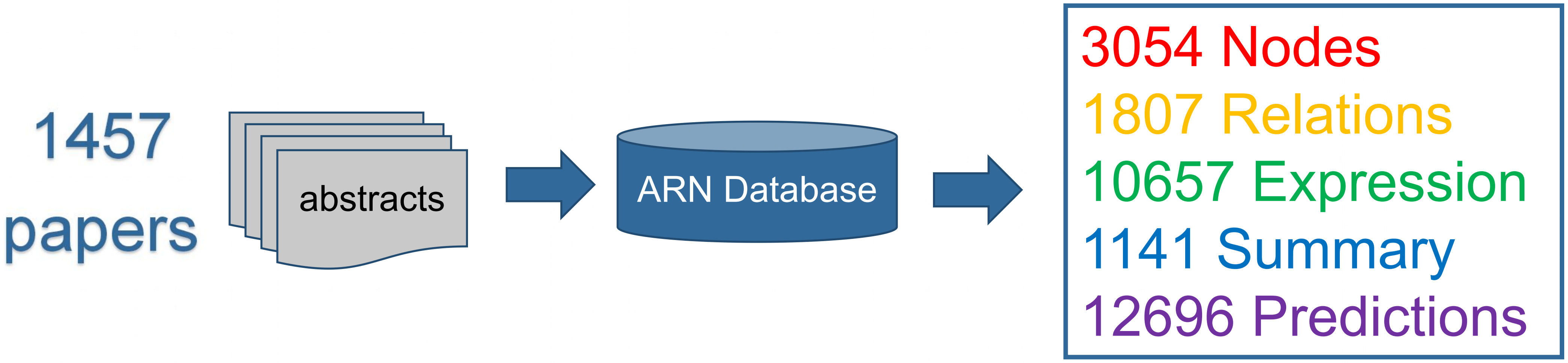

Paper= Abstract + Material + Methods + Datas ......

ARN

Definitions

1. **Node：** genes or miRs regulate adipogenic differentiation
2. **Symbol：** Official Symbol of nodes in NCBI-gene
3. **link:** Relationship between the nodes
4. **IF(%)：** quantification the influence of node on adipogenesis
5. **Agreement:** quantification the reliability of predictions, 100 means verified

How to use ARN database

ARN

Example 1
basic search

Example 2
Node analysis

Example 3
Paper analysis

Example 4
problem oriented
Data analysis

Example 5
Customize
Data analysis

Level 1

Level 2

Level 3

Level 4

Level 5

Example 1 basic search

1

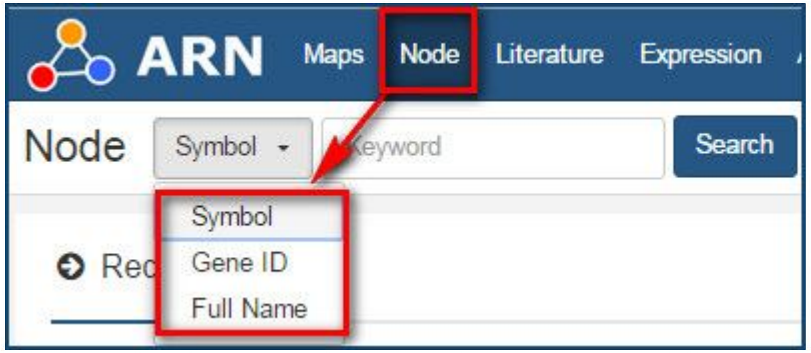

Node page

Users can search by node Symbol, Gene ID or full name

2

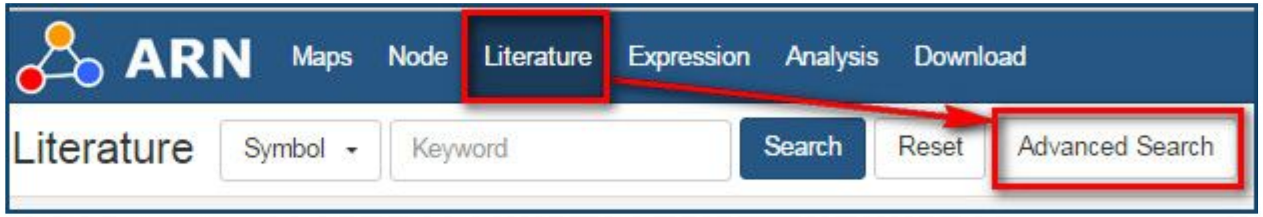

Literature page

The "advanced search" can screen articles according to the types of material, PMID or other details

Example 1 basic search

Screen the high throughput articles

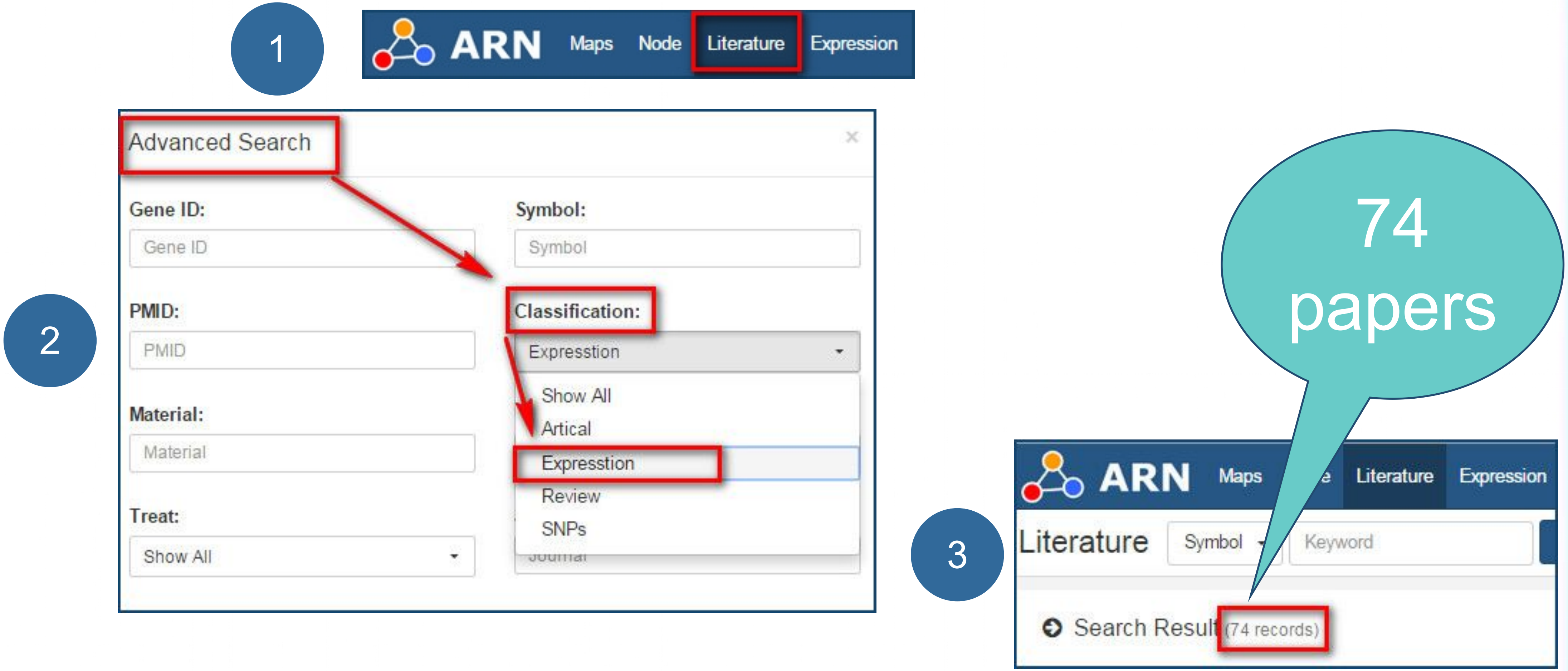

Example 2 Node analysis (Sp1)

ARN

1

ARN Maps **Node** Literature Expression

Node Symbol ▾ sp1 Search

2

Node Expression

#	PMID	Condition	Expression	Material
1	23894377	control VS cold-induced Liver (1 2)	(1 2.6574268)	mouse
2	19710927	osteogenic TF	H3K9AC Decreased	MSC

Cold induced Up-regulation

3

ARN Maps Node Literature Expression **Analysis** Download

See next page

Example 2 Node analysis (Sp1)

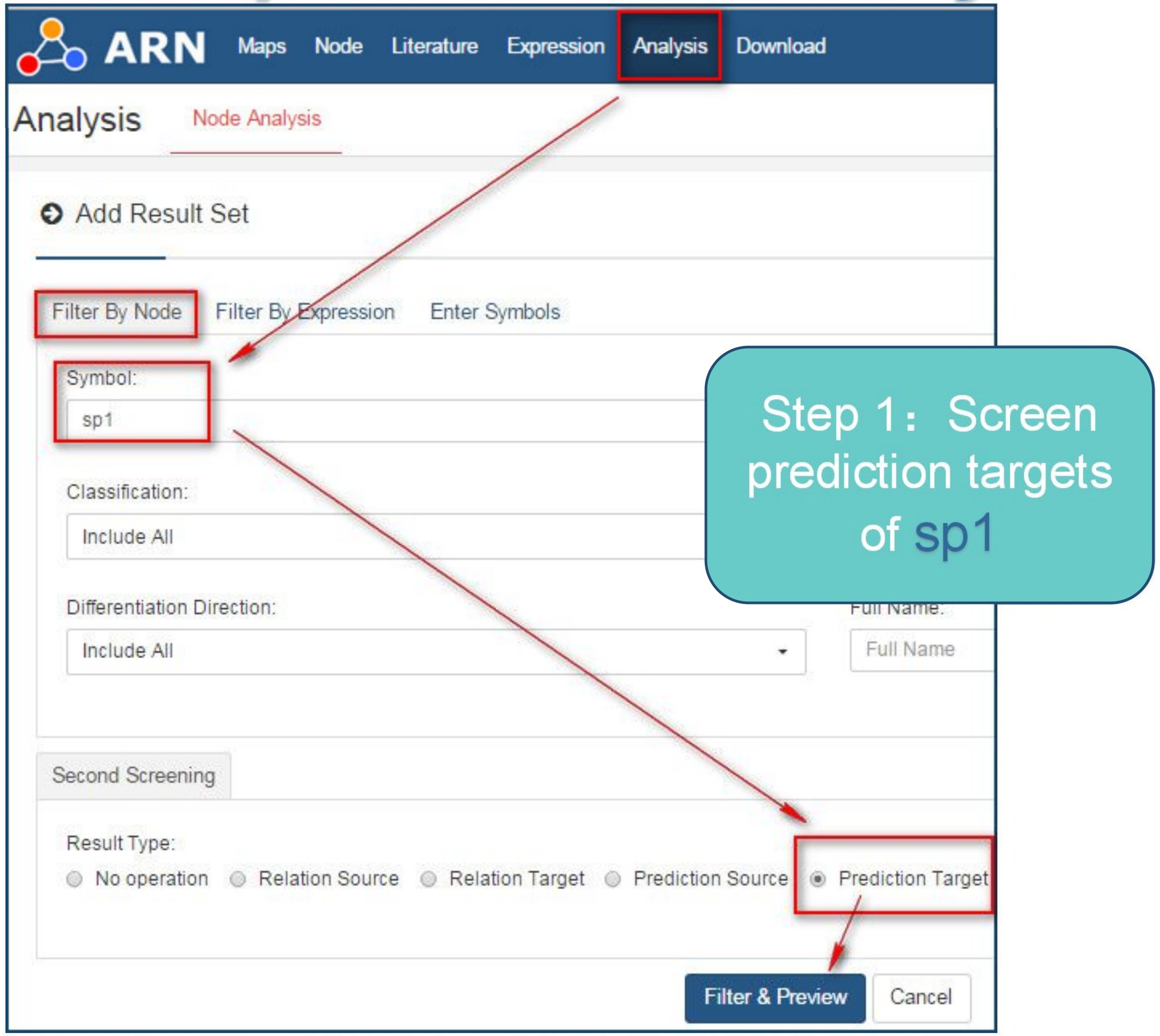

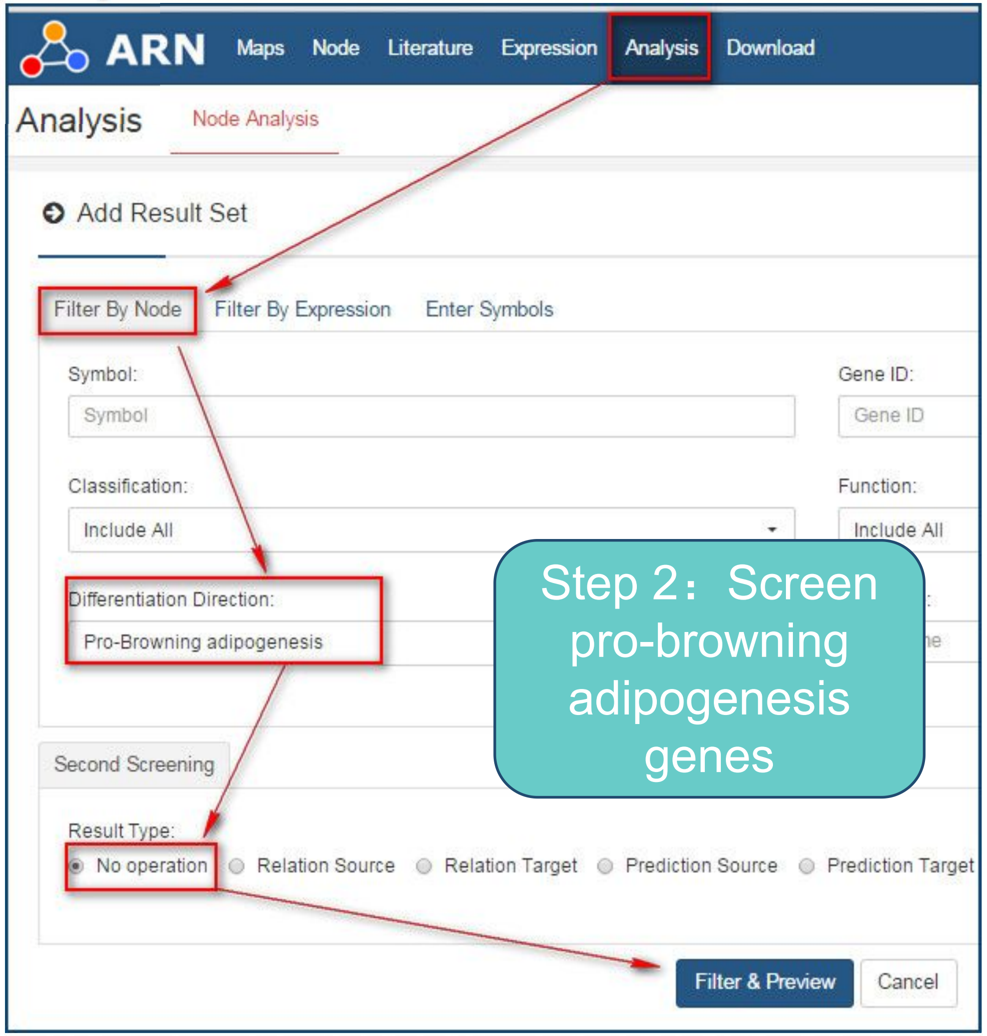

Example 2 Node analysis (Sp1)

ARN

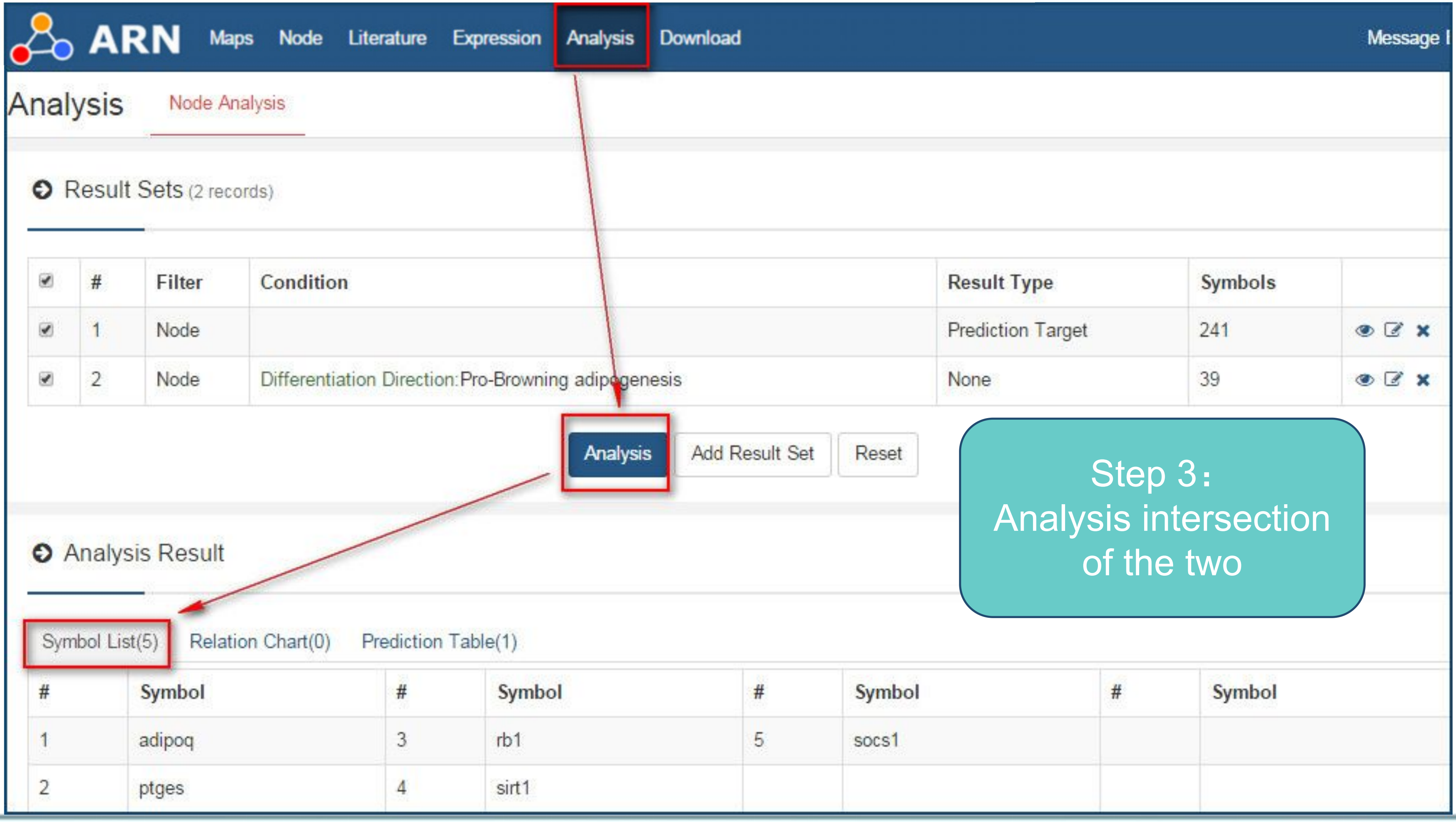

Step 3：
Analysis intersection of the two

Example 3: Paper analysis

Step 1：Screen results of paper (PMID 22496873)

Step 2：Select mirn30a for analysis

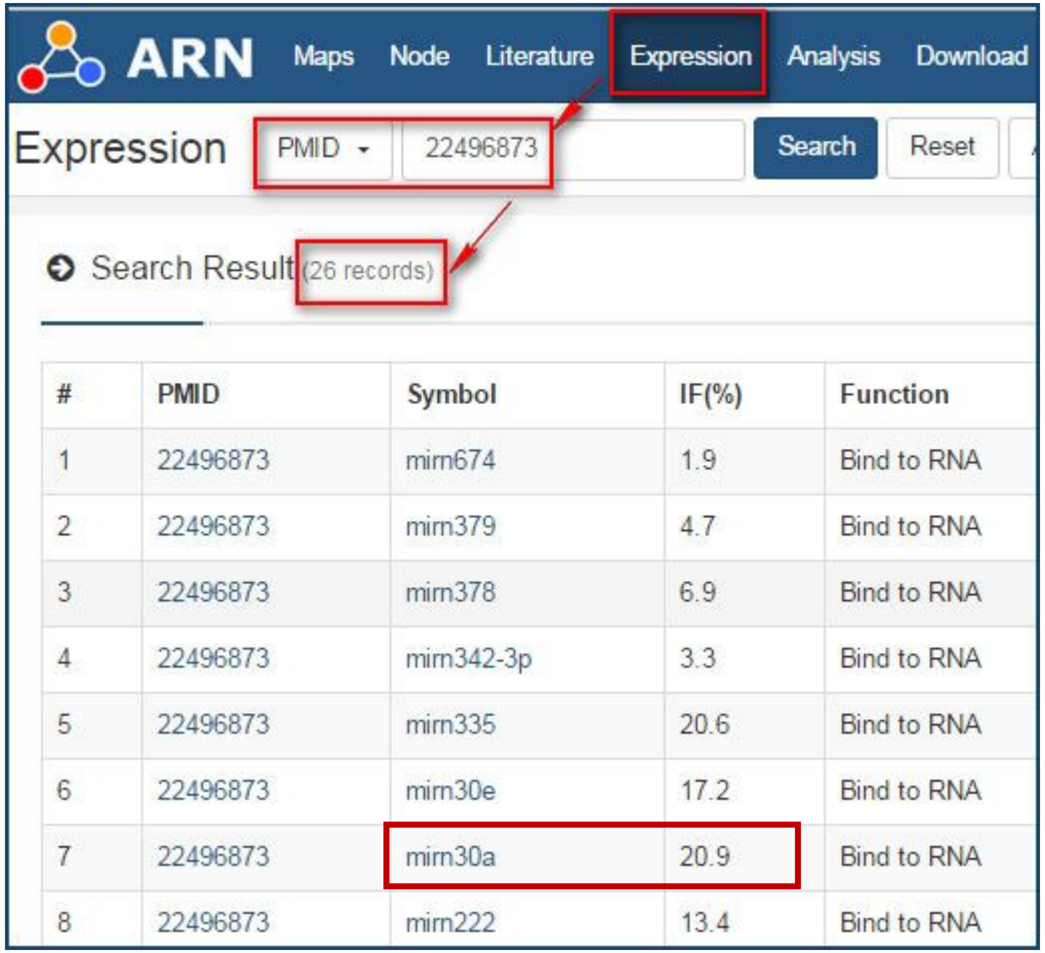

#	PMID	Symbol	IF(%)	Function
1	22496873	mirn674	1.9	Bind to RNA
2	22496873	mirn379	4.7	Bind to RNA
3	22496873	mirn378	6.9	Bind to RNA
4	22496873	mirn342-3p	3.3	Bind to RNA
5	22496873	mirn335	20.6	Bind to RNA
6	22496873	mirn30e	17.2	Bind to RNA
7	22496873	mirn30a	20.9	Bind to RNA
8	22496873	mirn222	13.4	Bind to RNA

Example 3: Paper analysis
Analyse mirn30a

Step 3：
Analyse the expression
of mirn30a

Step 4：
"Analysis" see the next page

ARN Maps Node Literature Expression

Osteo-dif down regulated
Adipo-dif up regulated

Node Expression

#	PMID	Condition	Expression
1	18784367	BMP2 induced C2C12 Osteo-Dif (0 2 4 8 1[illegible]	(2 1 1.5 -1 -3)
2	24307698	spindle-shaped (SS) VS round-shap[illegible] (RS) hMSCs(1 2)	(-1.8 2.8)
3	23564456	BMP2 induced C2C12 osteo-dif(0 8)	(1 0.4680)
4	20492721	MDI VS Li+MDI 3T3-L1 (1 2)	(2.36 1)
5	26537990	nonalcoholic fatty liver mice (1 2)	（0 -0.41)
6	26001136	hMSC VS Adipocyte(1 2)	(-2 2)
7	21767385	undif VS dif hADSC (1 2)	(1 3)
8	25356868	hMSCs Adipo-Dif (0 13)day	(0 1.4)
9	25751060	mice BMSCs 3mo VS 18 mo (3 18)	(3 -3)
10	19188425	Obesity VS nonObesity (1 2)	log2(-1 0)

Example 3: Paper analysis
Analyse mirn30a

ARN Maps Node Literature Expression Analysis Download

Analysis Node Analysis

Result Sets (3 records)

#	Filter	Condition	Result Type	Symbols
1	Node		Prediction Target	39
2	Node	Differentiation Direction:Pro-osteoblastogenesis	None	211
3	Node	Differentiation Direction:Anti-aidpogenesis	None	173

Step 5：Analysis

Step 6：Analysis intersection

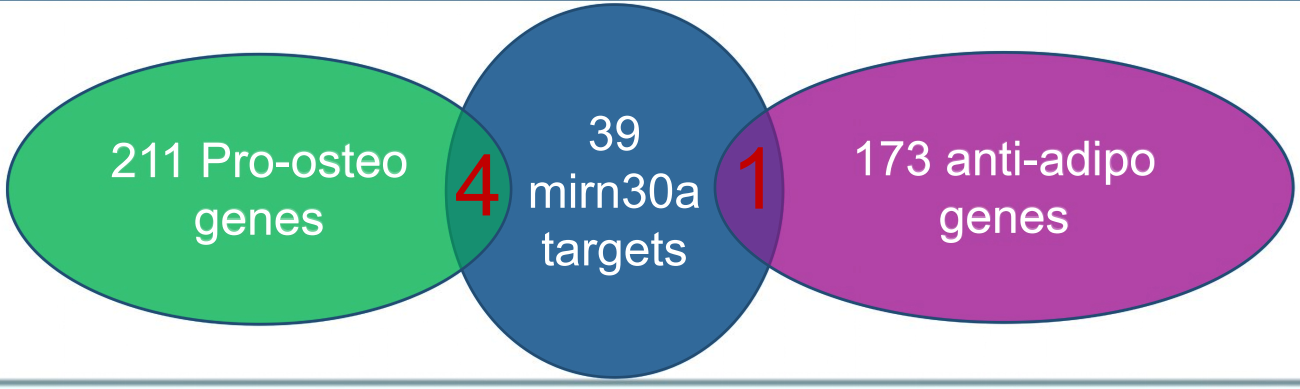

Example 3: Paper analysis
Analyse mirn30a

Step 7：Results

Source	target	IF(%)	分化方向
mirn30a	tsc22d3	12.3	抗成脂
	cbfb	4.9	促进成骨
	sox12	1.2	
	tsc22d3	12.3	
	wnt5a	5.3	

Step 8：Design verification test

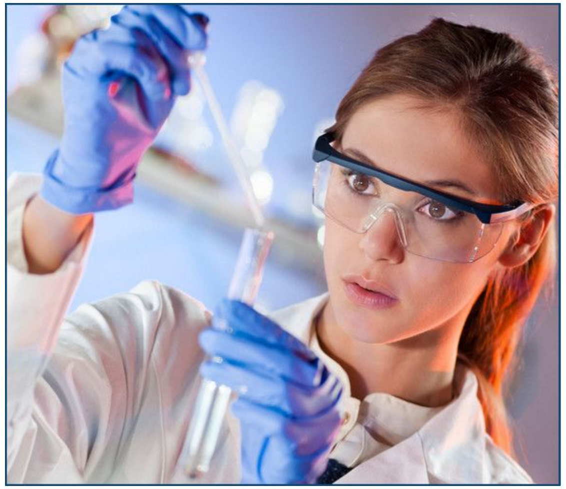

Example 4 problem oriented Data analysis

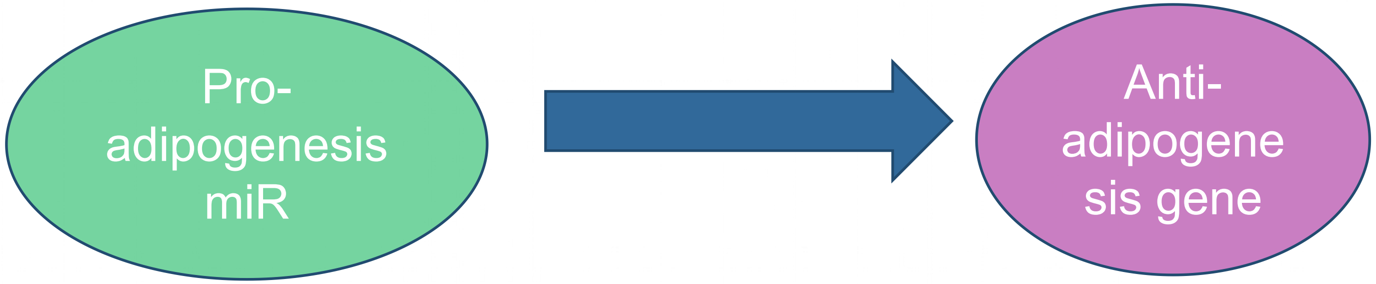

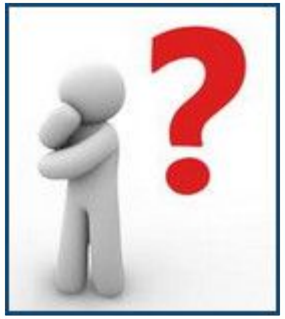

Is Pro-adipogenesis miR
promote adipogenesis
by regulating anti-adipogenesis gene?

Example 4: problem oriented Data analysis

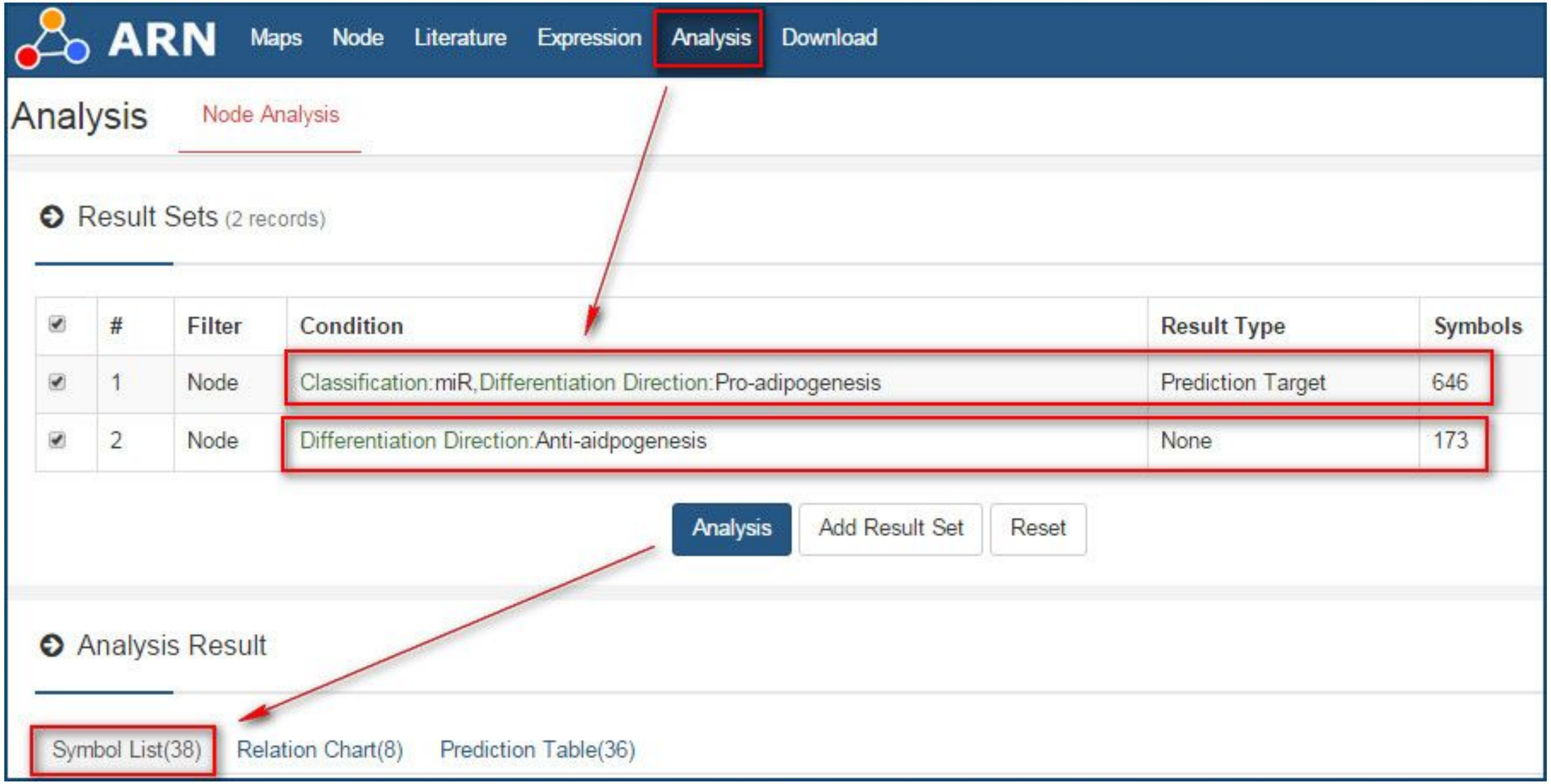

Example 5 Customize Data analysis

How to screen the adipogenic related genes in short time?

Table 1

List of differential expressed genes (DEGs) with P value ≤ 0.05 and fold changed ≥ 1.3 from microarray analysis.

145 up-regulated genes	85 down-regulated genes
1110059M19Rik, Acta1, Has1, Ccl7, Snrpn, Ndn, Ccl2, Tmem45b, Sfrp4, Xlr3c, Thbs1, Bmp3, Sfrp5, Smurf, Xlr4b, Ctgf, Timp1, Pamr1, Grb14, Cela1, Egr2, Duoxa1, Adamtsl2, Dact2, Cryab, Krt79, Cd44, Gfpt2, Sirpb1b, Slc1a1, Atp10a, Pdlim7, Spink2, Npr3, Arid5a, Krt1, Bhlhe40, Wnt2, Chsy1, Nol3, Socs3, F10, Timp4, Sct, Mgam, Pthlh, Synpo2, Rassf4, Tnfaip6, Mutyh, Mkrn3, Dnm1, Ccl5, Slc16a3, Pdlim1, Fbn1, Tph2, Dpysl3, Uchl1, Ube3a, Ccl4, Lep, LOC100038947, Rasl11a, Cd8a, Fn1, Col14a1, Bmp7, Ldlr, Slc41a2, Kcnc1, Creb5, Esd, Vgll3, Prelp, Mag, Hdgfl1, Nuak2, Lsp1, Expi, Lbp, Pla1a, Inhbb, Sybu, Wnt11, Slc22a2, Mefv, C1qtnf7, Gamt, Has2, Lrrc25, Herc2, Efhd1, Ms4a6d, Tmem100, Dact1, Rnd1, Tnfrsf11b, Thy1, Lgmn, Cxcr7, Hist1h1c, Mafb, Ranbp3l, Dnajb13, Rgs14, Ttll10, Syp, Fcna, E030010A14Rik, Nov., Adh4, Anxa1, Pprc1, Nipal3, Ugt3a2, Prdm8, AI467606, Emilin2, Smg6, Tspan17, Myo1g, Vdr, S100a6, Maff, Vcan, Sncg, Batf, Pcolce2, Apbb1ip, Cdkn1a, Mtap7, Itgam, Lrrc20, 1700019D03Rik, a, Pdlim4, Fxyd5, Csf1, Cmtm7, Myh2, Peli1, Nek6, Cd6, Ccbp2	Fndc5, Mmd2, [illegible] Ms4a1, Spon1, H2-[illegible] Tst, T[illegible]gds, Serpina3b, Hpca, Ly6d, Sfrp1, Cd79b, Apod, Faim3, Pappa, Acacb, Prnd, 1500015O10Rik, Cpxm2, Nmb, Sox9, Agpat9, Slc5a6, Dennd2d, 4930524B15Rik, 2900062L11Rik, Igfbp2, Ppargc1a, Avpr1a, Drd1a, Btnl9, Tbx3, Lrig1, Rnf144b, Gulp1, Aplnr, Ly6g6e, Ppargc1b, Gm7455, Pdgfrl, Dio3, Smpd3, Lrrn1, Itm2a, Bcat2, Slc27a1, Prlr, Arl4a, Lpin1, 2010001M09Rik, Vnn3, Celf6, Rhbg, Trim9, Mogat1, Slc44a5, Chdh, Dock9, Pcsk6, BC031353, Il15ra, Acsl1, Gdf10, Rab15, Mlxipl, Ppa1, Acot1, Alas1, Penk, Crispld2, Rcl1, Gria1, Nrg4, Baiap2l2, Itpka, Aspg, Slc22a23, Spink8, Col6a6, Mgst2, Bmf, Klhl2

Liu X, Tamada K. etc. Transcriptome profiling of white adipose tissue in a mouse model for 15q duplication syndrome. Genom Data. 2015 Jul 10;5:394-6.

Example 5 Customize Data analysis

Step 1: obetined 47 nodes

↓

Step 2: Query in ARN one by one

↓

Step 3: Design verification test

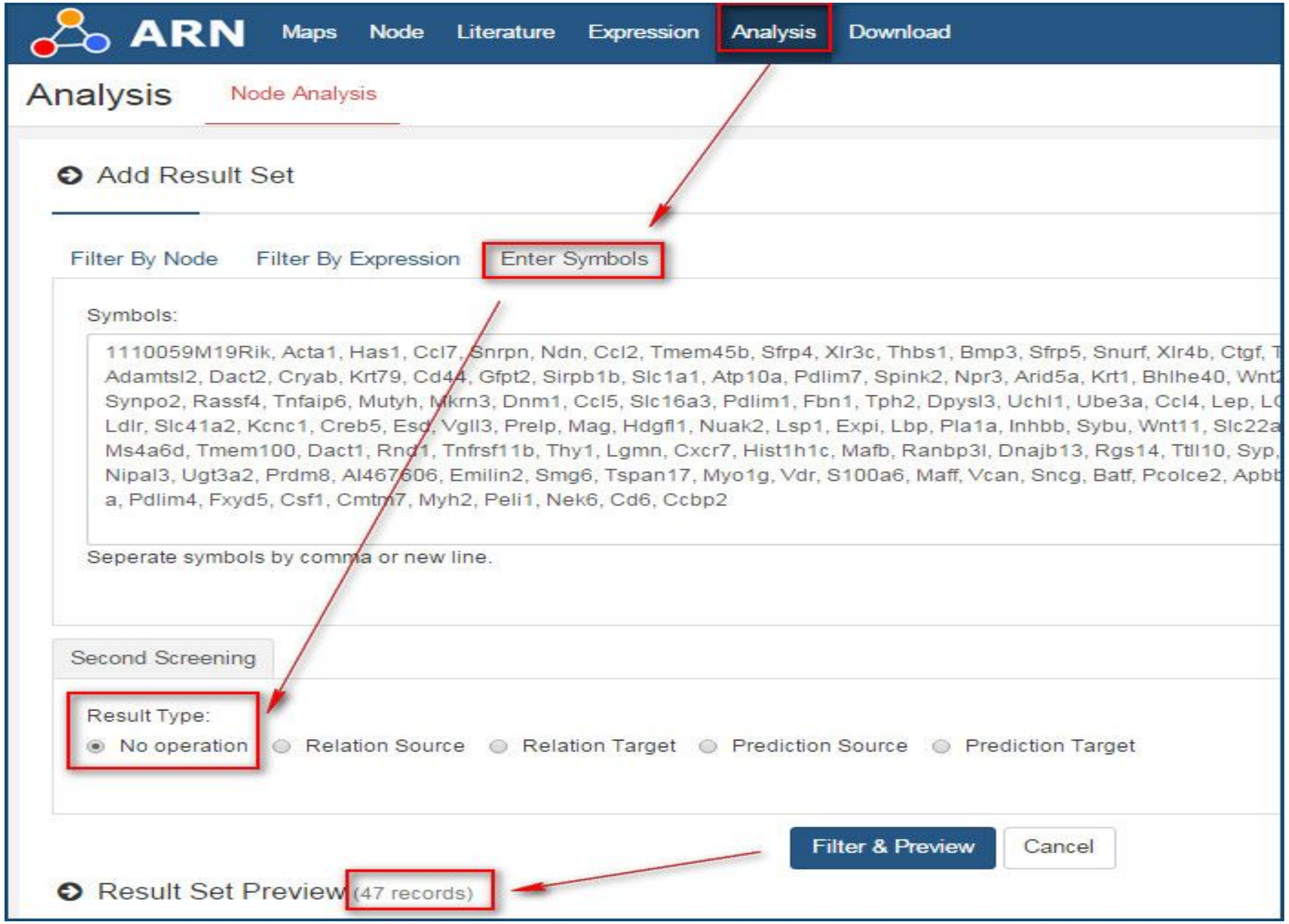

Welcome

to ARN
